# Effectiveness of Nitrous Oxide Sedation on Child’s Anxiety and Parent Perception During Inferior Alveolar Nerve Block: A Randomized Controlled Trial

**DOI:** 10.7759/cureus.48646

**Published:** 2023-11-11

**Authors:** Divya Mukundan, Deepa Gurunathan

**Affiliations:** 1 Department of Pediatric and Preventive Dentistry, Saveetha Dental College and Hospitals, Saveetha Institute of Medical and Technical Sciences, Saveetha University, Chennai, IND

**Keywords:** nitrous oxide oxygen sedation, less pain, pediatric dentistry, conscious sedation, anxiety

## Abstract

Introduction

Dental anxiety is a common phenomenon among children and can have significant implications for their overall oral health and well-being. Among the various dental procedures that induce anxiety in pediatric patients, the Inferior Alveolar Nerve Block (IANB) stands out as one of the most feared due to its perceived pain and discomfort. Dental anxiety not only affects the child's cooperation during the procedure but can also lead to long-lasting negative perceptions of dental care, resulting in the avoidance of necessary treatments in the future. Nitrous oxide (N_2_O) sedation is a well-established sedation technique in dentistry, widely used to manage anxiety and discomfort during dental procedures. However, its efficacy in reducing anxiety during the administration of IANB to pediatric patients remains the subject of ongoing research. The administration of N_2_O sedation during IANB may not only alleviate the child's anxiety but also influence the parent's perception of the procedure, which can have additional effects on the child's dental experience and future adherence to dental care. The aim of this study was to evaluate the effectiveness of N_2_O sedation on pain and anxiety in children before and after IANB administration and the parent's perceptions of sedation following the procedure.

Methods

The current study was a single-centered, double-blinded, randomized controlled trial. The participants were assigned randomly to two groups, with each group consisting of 20 participants. Group 1 (n = 20) was given only oxygen, and Group 2 (n = 20) was given N_2_O for sedation. Pain perception for local anesthesia was evaluated using the Face, Legs, Activity, Cry, Consolability scale. The anxiety of children was evaluated using the Facial Image scale. Parent satisfaction was analyzed using the Likert scale. Data were extracted before and after the procedure using the Mann-Whitney U test.

Results

Pain perception evaluated with the FLACC scale showed statistically low pain perception in Group 2 after the procedure with a p-value of 0.001, and anxiety levels assessed with FIS showed a significant difference in Group 2 after the procedure with a p-value of 0.003. Parent satisfaction was analyzed using the Likert scale, and Group 2 showed a statistically significant difference with a p-value of 0.001 after the procedure.

Conclusion

The administration of the N_2_O sedation results in a notable reduction in anxiety levels and pain perception, as well as better parental satisfaction. This method allows for a practically pain-free and anxiety-free environment.

## Introduction

Dental fear and anxiety are prevalent factors affecting dental healthcare in people of all ages, but they seem to manifest mostly in childhood and adolescence [1]. The incidence of dental anxiety in children and adolescents ranges from 5% to over 24% worldwide [2]. It has been observed that dental anxiety in children frequently leads to increasing levels of caries and behavioral management challenges. Children are most afraid of injections when they go to the dentist; approximately 12% of pediatric dental patients have reported inadequate local anesthesia, resulting in an increased demand for additional pain relief and modifications to anxiety management [3].

Anxiety can be treated pharmacologically or non-pharmacologically. Pharmacological management techniques include conscious sedation or general anesthesia, and non-pharmacological behavior control techniques include the tell show do technique, distraction, role modeling, positive reinforcement, and hypnosis [4,5]. The majority of dental procedures may be carried out using nonpharmacological behavior modification techniques; however, in the case of highly anxious children, pharmacological management may be necessary for a successful treatment that includes conscious sedation, which is delivered by a variety of combinations and general anesthesia [6,7].

Conscious sedation has proven to be a valuable method for practitioners, providing a safer alternative to general anesthesia whenever possible, according to Mourad et al. [8]. According to the Council of European Dentists [9], Nitrous oxide (N_2_O) sedation is currently “the standard sedative technique” in pediatric dentistry. There are many benefits to N_2_O sedation, which include rapid onset of action, minimal reflex impairment, and rapid postoperative recovery within five minutes [8]. Numerous studies have highlighted the advantages of N_2_O sedation in reducing anxiety and improving the dental experience for children undergoing various procedures [10-12]. Similarly, the most commonly used injection technique for achieving pulpal anesthesia in primary mandibular teeth is the inferior alveolar nerve block (IANB), and it has been reported that IANB is associated with pain and discomfort [13,14]. However, there is a lack of studies directly comparing N_2_O sedation and IANB in terms of their efficacy in reducing anxiety and managing pain. Hence, the present study compared the efficacy of N_2_O sedation in reducing children's anxiety and pain perceptions before and after IANB administration and parents' perceptions after the procedure.

## Materials and methods

Study design

**Figure 1:**
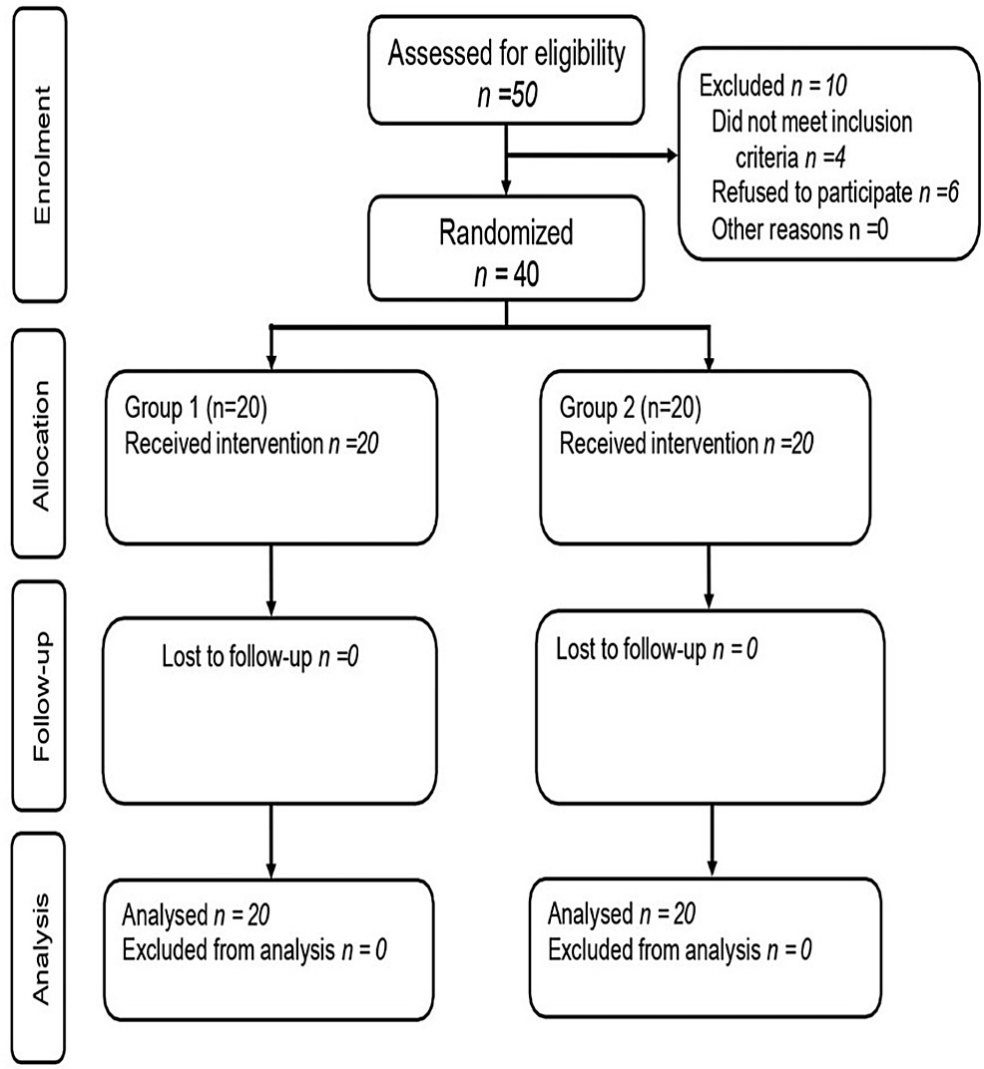
Consort flow diagram showing the number of participants through each stage of the randomized controlled trial.

The current study was a double-blinded, randomized controlled trial. Forty children were allotted randomly into two groups of 20 each. A computer-generated series of random numbers was used to split the participants into groups based on inclusion criteria. The timeline of the randomized controlled trial is shown in Figure 1.

Study Setting 

The study was conducted in the Department of Pediatric and Preventive Dentistry of a private dental college in Chennai.

Ethical Clearance

Prior to the beginning of the study, the study was approved by the institutional ethical committee (SRB/SDMDS07/19PEDO/24) and registered in the Clinical Trials Registry-India (CTRI/2019/09/021381). Parents provided written informed consent. The anonymity of the participants was maintained.

Study Population

The study population included patients visiting the outpatient Department of Pediatric and Preventive Dentistry aged 6-9 years from March 2020 to June 2021.

Inclusion Criteria

Children who required Dental treatment requiring IANB anesthesia, participants who belong to Frankl 2 and 3 ratings during the examination process, participants with no prior dental experience and participants belonging to the American Society of Anesthesiologists 1 category.

Exclusion Criteria

Children with clinical condition contraindicating the use of N_2_O sedation such as a cold and who were allergic to lignocaine.

Sample Size Calculation

The sample size was calculated by G Power based on the study by Baeder et al [11], with a p value of 0.05 and 95 power with an effect size of 0.636. The calculated sample size was 20.

Randomization

Participants were randomly allocated into two groups of 20 each. Group 1 (n = 20) was given only oxygen as a placebo, and Group 2 (n = 20) was given N_2_O for sedation. The participants and the statistical analyst were blinded during the procedure.

Treatment protocol

**Figure 2:**
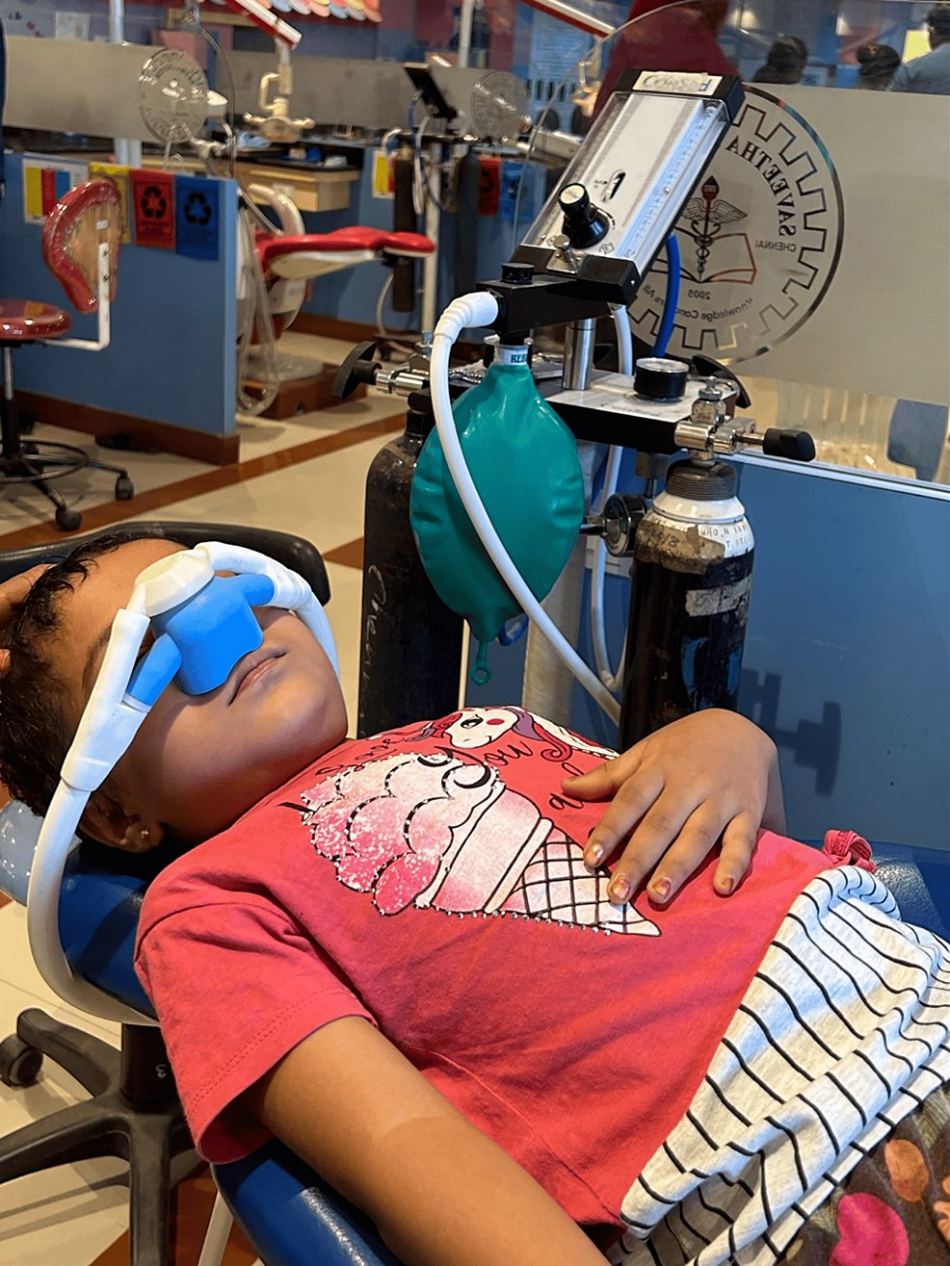
Participant being administered 100% oxygen

All participants received IANB for the extraction of mandibular molar were evaluated by the same postgraduate student, who also performed and continuously assessed the children’s cooperation. For the study, a portable compact device known as Consed (Consed International, Kerala, India) was used. Consed enables the uninterrupted supply of N_2_O and oxygen, with a flow control knob that regulates the combined flow of these gases [15]. In Group 1, the procedure begins with the administration of 100% oxygen at a flow rate of 1 L/minute for two to three minutes (Figure 2).

As the patient breathed, the reservoir bag was constantly monitored, and local anesthesia was slowly administered. During the course of the treatment, the child's ocular activity, overall responses, and level of consciousness were closely observed and assessed. Following the completion of administrating the LA, the concentration of oxygen was lowered.

**Figure 3:**
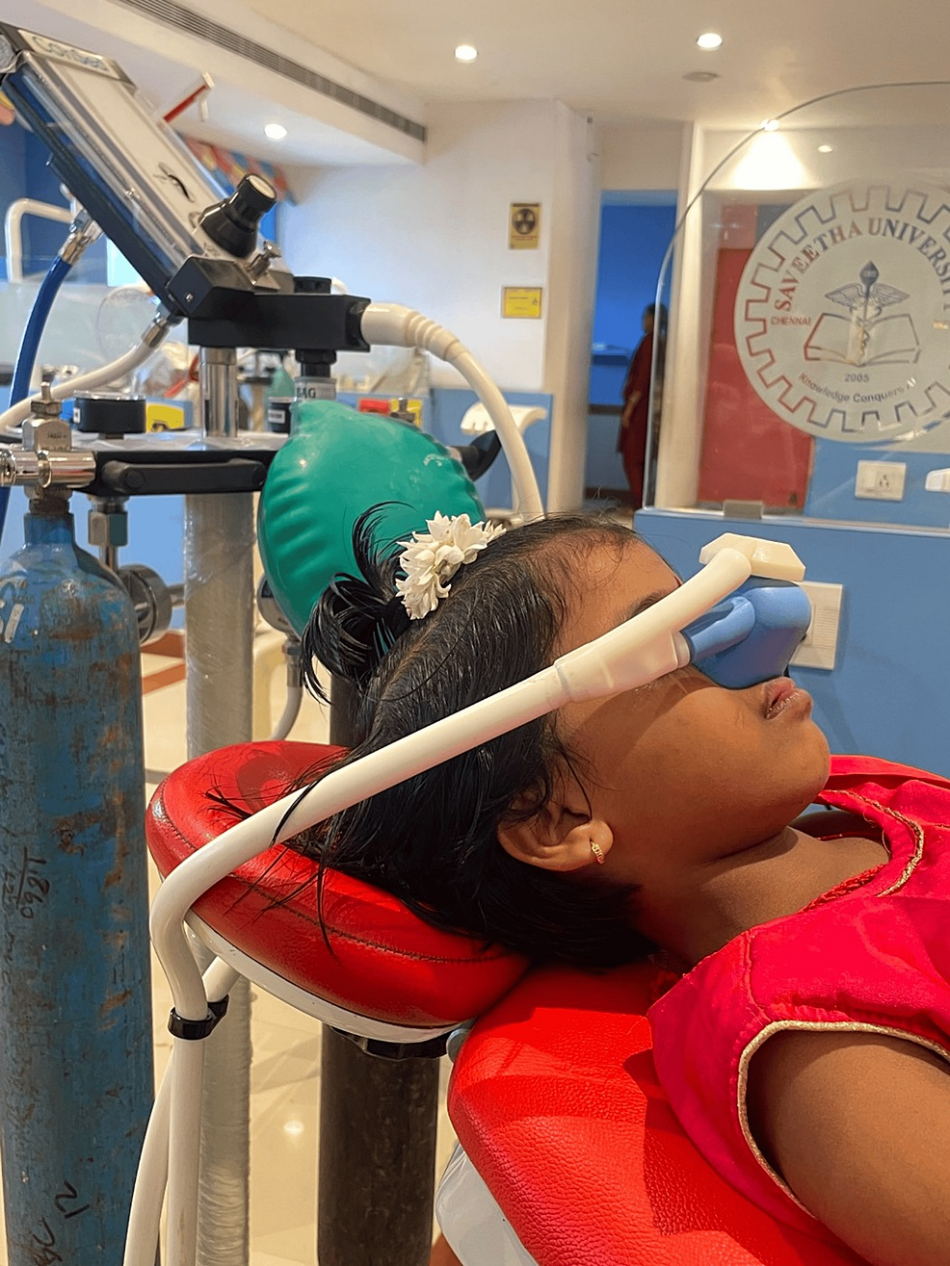
Participant being administered N2O sedation

In Group 2, the procedure began by administering 100% oxygen for a duration of two to three minutes with a flow rate of 1 L/minute. The monitoring of the reservoir bag was done in close proximity to the patient's respiration. The titration of N_2_O was done at 10% intervals, gradually increasing the concentration up to 50% (Figure 3).

Subsequently, the N_2_O concentration was maintained at this level. The local anesthesia was then administered slowly. During the course of the treatment, close observation was maintained of the child's ocular activity, overall responses, and level of consciousness including pulse rate and oxygen saturation. Following the completion of administrating the LA, the concentration of gas was reduced, thereby ensuring the continuous administration of 100% oxygen. 100% oxygen was administered for three to five minutes.

An IANB along with a lingual nerve block was administered after applying topical anesthetic gel (Progel B-Benzocaine Gel 20%) for both groups. A 30-gauge, 25 mm long needle (Hindustan syringes-Dispo van) was used to administer 1.5 mL of 2% lignocaine hydrochloride and 1:100,000 adrenaline. One millimeter per minute was the normal rate of infusion.

Anxiety and pain perception were analyzed before the procedure began and after the procedure was completed. Anxiety was analyzed using the FIS, which consists of a row of five faces ranging from extremely happy to extremely sad. The participants were instructed to point to the face they felt most like at the time. The scale is assessed by assigning a value of one to the most positively affective face and a value of five to the most negatively affective face. Pain perception was analyzed using the FLACC scale. There are five criteria on the scale, and each is given a score of 0, 1, or 2. The five categories, namely face, legs, activity, crying, and consolability, are each given a score between 0 and 2, adding up to a total between 0 and 10. Parent's satisfaction after the procedure was analyzed using the Likert scale. A score of 1-5 was recorded for both groups, with a score of 1 for very dissatisfied and a score of 5 for very satisfied.

Statistical analysis

The mean and standard deviation for anxiety, pain, and parent satisfaction for both groups were calculated using IBM SPSS Statistics for Windows, Version 22.0 (Released 2013; IBM Corp., Armonk, NY, United States). The final results of the Shapiro-Wilks and Kolmogorov-Smirnov tests for normality revealed that none of the variables followed a normal distribution. Consequently, a non-parametric approach was used to analyze the data. The data were compared between groups using the Mann Whitney U Test. The level of significance was set at 5% (or 0.05).

## Results

Forty participants were included in the study, out of which 17 participants were boys and 23 participants were girls. The demographic characteristics and the age distribution of the included children are given in Table 1.

**Table 1:** Demographic characteristics of the included children

Demographic Data	Group 1	Group 2
Age (Years)	7.5±0.7	8.4 ± 0.5
Male (%)	9 (45 %)	8 (40 %)
Female (%)	11 (55 %)	12 (60%)

Mann-Whitney U Test was done to compare the anxiety and pain perceptions between Group 1 and Group 2 before and after the procedure. There was a highly significant difference in the amount of pain before and after the procedure in children who received N_2_O sedation, whereas children who received only oxygen did not show any difference. Further, when postoperative pain assessment between two groups was carried out, group 2 children experienced less pain compared to group 1, clearly indicating the advantage of N_2_O in alleviating pain perception.

Anxiety was analyzed using the Facial Image Scale. Similar to pain perception, anxiety was significantly lessened after the procedure compared to before the procedure in group 2. Further, anxiety was substantially less after the procedure in Group 2 compared to Group 1, and Group 2 showed a statistically significant difference, with an improvement in their behavior after the procedure with a p-value of 0.003 (Table 2).

**Table 2:** Comparison of mean scores of pain and anxiety in Group 1 (oxygen) and Group 2 (N2O) using the Mann-Whitney U test

	Group 1			Group 2		
Outcome	Before the procedure (Mean±SD)	After the procedure (Mean±SD)	P-value	Before the procedure (Mean±SD)	After the procedure (Mean±SD)	P-value
Pain	2.65±0.18	2.45±0.68	0.563	2.6±0.59	0.25±0.44	0.001*
Anxiety	2.45±0.93	2.25±0.74	0.762	2.6±0.69	0.23±0.53	0.003*

Parent satisfaction was analyzed using the Likert scale after the procedure, where parents of children in group 2 showed a statistically significant higher degree of satisfaction (p=0.001) (Table 3).

**Table 3:** Comparison of mean scores of parental satisfaction in Group 1 (Oxygen) and Group 2 (N2O) using the Mann-Whitney U test

Outcome	Group 1 After the procedure (Mean±SD)	Group 2 After the procedure (Mean±SD)	P-value
				Parent satisfaction	0.35±0.48	4.75±0.44	0.001

## Discussion

Anxiety is one of the major challenges in pediatric dental treatment. The procedure that makes both children and adults most anxious is the administration of local anesthesia. Though local anesthesia results in further painless treatment, it also makes young patients very anxious [16]. The behavioral management of anxious patients requiring extensive dental care is often enhanced with the use of sedation. The inhalation route for administering sedatives is the most convenient for children and the most popular among pediatric dentists. Hence, the current study compared the effectiveness of N_2_O sedation in lowering children's anxiety and pain before and after IANB administration and parent satisfaction after the procedure.

In the present study, the N_2_O group's anxiety levels in children were much lower when compared to Group 1. N_2_O triggers its analgesic action by inhibiting N-methyl-D-aspartate (NMDA) glutamate receptors and releasing endogenous opiate peptides, which then activate opioid receptors. There are three enzymes that mediate the anxiolytic effects of N_2_O are nitric oxide synthase, soluble guanylyl cyclase, and cyclic guanosine monophosphate-dependent protein kinase. N_2_O produces the necessary analgesic effect by suppressing the excitatory response that NMDA usually elicits in the nervous system. Gamma-aminobutyric acid is activated via the binding site, which results in the anxiolytic action [10].

N_2_O sedation is currently used in the pediatric emergency department to manage pain during a variety of procedures, such as fracture reduction and laceration repair. It has been observed that children undergoing even minor procedures, such as foreign body removal and abscess drainage, significantly lessen their pain and anxiety when N_2_O is administered [17]. The findings of the study indicate that the administration of N_2_O sedation has a positive impact on mood enhancement and the reduction of dysphoric symptoms across all patients. The results of the present study were in accordance with the findings of Zacny et al. [18], who examined the effects of nitrous gas sedation on patients with different levels of preoperative dental anxiety. 

Pain perception was analyzed using the FLACC scale, which has great validity and reliability. There are five criteria on the scale, and each is given a score of 0, 1, or 2. A significant difference was found between Group 1 and Group 2, and this was in accordance with the study done by Jacobs et al. [16], who examined the analgesic effects of N_2_O during three different types of mandibular block procedures. Frankl behavior rating was used to assess the child's behavior in this study. Frankl rating has been shown to be effective in clinical practice, with a direct relationship between predicted and actual manifested behavior during dental appointments [19].

Prior to the administration of sedation, parents were given extensive information, ensuring that they were adequately informed of the procedural details. A significant proportion of parents expressed satisfaction with the efficacy of sedation, demonstrating its efficiency in fulfilling its intended objective while inflicting minimum psychological discomfort on their children. The results of the present study were in accordance with the findings of Akr et al. [20], who assessed preschool children’s satisfaction after treatment under N_2_O sedation.

The main complications related to pediatric conscious sedation are hypoxia, nausea, and vomiting [21]. However, this study was not powered to examine this possible side effect. The results of this study further support the fact that 90% of children receiving IANB for dental treatments were able to successfully complete their treatment under sedation, with N_2_O sedation level at 50%. Deep drowsiness or effects similar to general anesthesia could result from a higher dose of N_2_O [22].

Strength and limitation of study

The benefit of conducting a double-blind, randomized controlled trial for N_2_O sedation is that it reduces bias and ensures that results can be directly related to the treatment, which results in exceptionally strong scientific evidence. The strength and unique addition of the present study is the inclusion of parental perception, which acknowledges their pivotal role in a child's dental experience and ensures a comprehensive assessment. The study's result has shown N_2_O sedation in reducing the emotional stress associated with dental injections There were a few limitations in the current investigation such that the inclusion of equal numbers of male and female participants will enable to evaluate the perception of pain in a better manner.

## Conclusions

N_2_O sedation is an effective option for treatment under local anesthesia to alleviate pain, reduce anxiety, and improve parent satisfaction. This method allows for a practically pain-free and anxiety-free environment.

Thus, this study highlights the beneficial effects of inhaled N_2_O sedation as a suitable option for anxious children undergoing dental treatment. Further, N_2_O sedation is a secure and effective way for pediatric patients to manage their anxiety and improve their entire dental experience. The incorporation of N_2_O sedation into clinical practice can be a valuable asset for healthcare professionals seeking to optimize patient comfort and well-being during procedures.

## References

[1] Klingberg G, Broberg AG (2007). Dental fear/anxiety and dental behaviour management problems in children and adolescents: a review of prevalence and concomitant psychological factors. Int J Paediatr Dent.

[2] Popescu SM, Dascălu IT, Scrieciu M, Mercuţ V, Moraru I, Ţuculină MJ (2014). Dental anxiety and its association with behavioral factors in children. Curr Health Sci J.

[3] Nakai Y, Milgrom P, Mancl L, Coldwell SE, Domoto PK, Ramsay DS (2000). Effectiveness of local anesthesia in pediatric dental practice. J Am Dent Assoc.

[4] Kotian N, Subramanian EMG, Ravindran V (2020). Video modelling technique used to manage the behaviour of uncooperative children in a dental set up. Braz Dent Sci.

[5] Cianetti S, Paglia L, Gatto R, Montedori A, Lupatelli E (2017). Evidence of pharmacological and non-pharmacological interventions for the management of dental fear in paediatric dentistry: a systematic review protocol. BMJ Open.

[6] Oubenyahya H, Bouhabba N (2019). General anesthesia in the management of early childhood caries: an overview. J Dent Anesth Pain Med.

[7] Wilson S, Alcaino EA (2011). Survey on sedation in paediatric dentistry: a global perspective. Int J Paediatr Dent.

[8] Mourad MS, Splieth CH, Al Masri A, Schmoeckel J (2022). Potential for nitrous oxide sedation in pedodontics practice to reduce the need for dental general anesthesia. Quintessence Int.

[9] (2019). CED Resolution on the Use of Nitrous Oxide Inhalation Sedation. https://www.omd.pt/content/uploads/2019/12/CED-DOC-2019-055-E.pdf.

[10] Kharouba J, Somri M, Hadjittofi C, Hasan J, Blumer S (2020). Effectiveness and safety of nitrous oxide as a sedative agent at 60% and 70% compared to 50% concentration in pediatric dentistry setting. J Clin Pediatr Dent.

[11] M Baeder F, F Silva D, Cl de Albuquerque A, Tbr Santos M (2017). Conscious sedation with nitrous oxide to control stress during dental treatment in patients with cerebral palsy: an experimental clinical trial. Int J Clin Pediatr Dent.

[12] Nazzal H, El Shahawy OI, Al-Jundi S, Hussein I, Tahmassebi JF (2021). The use of behaviour management techniques amongst paediatric dentists working in the Arabian region: a cross-sectional survey study. Eur Arch Paediatr Dent.

[13] Alzahrani F, Duggal MS, Munyombwe T, Tahmassebi JF (2018). Anaesthetic efficacy of 4% articaine and 2% lidocaine for extraction and pulpotomy of mandibular primary molars: an equivalence parallel prospective randomized controlled trial. Int J Paediatr Dent.

[14] Jeevanandan G, Juliet S, Govindaraju L, Ravindran V, Subramanian E (2020). Comparison between three rotary files on quality of obturation and instrumentation time in primary teeth − A double blinded randomized controlled trial. J Orofac Sci.

[15] (2023). India’s best dental conscious sedation machines manufactures. https://consedinternational.com/contact-conscious-sedation/.

[16] Jacobs S, Haas DA, Meechan JG, May S (2003). Injection pain: comparison of three mandibular block techniques and modulation by nitrous oxide:oxygen. J Am Dent Assoc.

[17] Duarte LTD, Duval Neto GF, Mendes FF (2012). Nitrous oxide use in children. Rev Bras Anestesiol.

[18] Zacny JP, Hurst RJ, Graham L, Janiszewski DJ (2002). Preoperative dental anxiety and mood changes during nitrous oxide inhalation. J Am Dent Assoc.

[19] Sivakumar P, Gurunathan D (2019). Behavior of children toward various dental procedures. Int J Clin Pediatr Dent.

[20] Akr SP, Mungara J, Vijayakumar P, Murali G, Veerapandian A (2022). Assessment of effectiveness acceptability complications and parental satisfaction of pediatric dental patients treated under nitrous oxide-oxygen inhalational sedation using Porter silhouette mask. Int J Clin Pediatr Dent.

[21] Mohan R, Asir VD, Shanmugapriyan Shanmugapriyan, Ebenezr V, Dakir A, Balakrishnan Balakrishnan, Jacob J (2015). Nitrousoxide as a conscious sedative in minor oral surgical procedure. J Pharm Bioallied Sci.

[22] Haider K, Mittal N, Srivastava B, Gupta N (2022). A double-blind randomized controlled trial to compare the safety and efficacy of dexmedetomidine alone and in combination with ketamine in uncooperative and anxious paediatric dental patients requiring pulpectomy. Eur Arch Paediatr Dent.

